# Activating Transcription Factor 3 regulates in part the enhanced tumour cell cytotoxicity of the histone deacetylase inhibitor M344 and cisplatin in combination

**DOI:** 10.1186/1475-2867-10-32

**Published:** 2010-09-09

**Authors:** Carly St Germain, Anna O'Brien, Jim Dimitroulakos

**Affiliations:** 1 Centre for Cancer Therapeutics, Ottawa Hospital Research Institute, 501 Smyth Road, Ottawa, K1H 8L6, Canada; 2 Department of Medicine, the University of Ottawa, 451 Smyth Road, Ottawa, K1H 8M5, Canada; 3 Department of Biochemistry, the University of Ottawa, 451 Smyth Road, Ottawa, K1H 8M5, Canada

## Abstract

**Background:**

Activating Transcription Factor (ATF) 3 is a key regulator of the cellular integrated stress response whose expression has also been correlated with pro-apoptotic activities in tumour cell models. Combination treatments with chemotherapeutic drugs, such as cisplatin, and histone deacetylase (HDAC) inhibitors have been demonstrated to enhance tumour cell cytotoxicity. We recently demonstrated a role for ATF3 in regulating cisplatin-induced apoptosis and others have shown that HDAC inhibition can also induce cellular stress. In this study, we evaluated the role of ATF3 in regulating the co-operative cytotoxicity of cisplatin in combination with an HDAC inhibitor.

**Results:**

The HDAC inhibitor M344 induced ATF3 expression at the protein and mRNA level in a panel of human derived cancer cell lines as determined by Western blot and quantitative RT-PCR analyses. Combination treatment with M344 and cisplatin lead to increased induction of ATF3 compared with cisplatin alone. Utilizing the MTT cell viability assay, M344 treatments also enhanced the cytotoxic effects of cisplatin in these cancer cell lines. The mechanism of ATF3 induction by M344 was found to be independent of MAPKinase pathways and dependent on ATF4, a known regulator of ATF3 expression. ATF4 heterozygote (+/-) and knock out (-/-) mouse embryonic fibroblast (MEF) as well as chromatin immunoprecipitation (ChIP) assays were utilized in determining the mechanistic induction of ATF3 by M344. We also demonstrated that ATF3 regulates the enhanced cytotoxicity of M344 in combination with cisplatin as evidenced by attenuation of cytotoxicity in shRNAs targeting ATF3 expressing cells.

**Conclusion:**

This study identifies the pro-apoptotic factor, ATF3 as a novel target of M344, as well as a mediator of the co-operative effects of cisplatin and M344 induced tumour cell cytotoxicity.

## Background

Alteration of gene expression plays an a role in tumourigenesis and progression of cancer. Modulation of gene expression, for example, tumour suppressors or oncogenes, are not exclusively due to mutations and can be manipulated through transcriptional regulation mechanisms which include DNA methylation and histone modification [1]. In cancer cells, the balance between histone acetylation and deacetylation catalyzed by histone acetyltransferases and histone deacetylases (HDAC), respectively, is often disrupted. For example, altered expression and aberrant recruitment of HDACs have been reported in tumours [2]. HDACs catalyze the removal of acetyl groups from histones resulting in chromatin condensation and transcriptional repression [1,3,4]. HDAC inhibitors act to reverse this transcriptional silencing of genes, which include tumour suppressors [1,3,4]. HDAC inhibitors are generally small molecule inhibitors that can readily diffuse across cellular membranes and directly interact with the zinc ion at the base of the catalytic pocket of this enzyme blocking substrate interaction and activity [1]. Coupled with their ability to induce cell cycle arrest, apoptosis, and disruption of angiogenesis, HDAC inhibitors have been evaluated as cancer therapeutic agents [1,3,4]. Currently the HDAC inhibitor, vorinostat, has been FDA approved for clinical use for treatment against cutaneous T-cell lymphoma [5].

cis-Diamminedichloroplatinum(II) (cisplatin) is among the most active anti-tumour agent used in human chemotherapy and widely used in various tumour types including lung and ovarian cancers [6]. Acquired resistance and toxicities associated with treatment are major impediments inhibiting their efficacy [7]. Cisplatin is primarily considered as a DNA-damaging agent that forms various types of bi-functional adducts in reaction with cellular DNA [6]. Cisplatin becomes activated intra-cellularly by the aquation of one of two chloride leaving groups, and subsequently covalently binding to DNA, forming DNA adducts [8]. The final cellular outcome of DNA adduct formation is generally apoptotic cell death, thought to occur through halting of cellular processes such as replication and transcription leading to prolonged G2 phase cell-cycle arrest, and deregulation of signal transduction pathways involved in growth, differentiation, and stress responses [7]. Cellular mechanisms of resistance to platinum-based chemotherapeutics are multifactorial and contribute to severe limitation in their use in clinical practice. They include molecular events inhibiting drug-DNA interaction, such as a reduction in cisplatin accumulation inside cancer cells or inactivation by thiol-containing species [6]. Other mechanisms of resistance acting downstream to the initial reaction of cisplatin with DNA, include an increase in adduct repair and a decrease in induction of apoptosis [7].

Pre-clinical and clinical studies have demonstrated that HDAC inhibitors can enhance the anticancer activity of a variety of epigenetic as well as chemotherapeutic agents including cisplatin [2,9]. For example, promising clinical trials combining platins as well as other chemotherapeutics with HDAC inhibitors have been conducted [10,11]. The ability of HDAC inhibitors to enhance the anti-cancer activity of known chemotherapeutic drugs is believed to be related to their function as positive regulators of gene transcription. As such, HDAC inhibitors have pleiotropic effects and can alter the expression of a wide variety (1000 s) of genes [1-3]. In particular, HDAC inhibitor treatment has been shown to augment expression of genes such as cell cycle suppressor, p21, apoptotic factors related to both extrinsic (death receptors and ligands) and intrinsic (Bcl-2 family members) pathways, and angiogenic factors such as HIF1α and VEGF [1-3].

It is well established that HDAC inhibitors can enhance the anticancer activity of cisplatin in vitro in a variety of cancer cell models [12-17]. Few studies exist, however, detailing the mechanism of enhanced anti-cancer effects by HDAC inhibitors in combination with cisplatin. For example, Rikiishi et al, correlated enhanced cytotoxicity by HDAC inhibitors in combination with cisplatin with reduced levels of the antioxidant intracellular reduced glutathione (GSH) in an oral squamous cell carcinoma model [18]. Our recent work has demonstrated that cisplatin treatment induces Activation of Transcription Factor (ATF) 3, a member of the ATF/cyclic AMP response element-binding family, regulates cisplatin-induced cytotoxicity [19]. ATF3 expression is induced by a wide variety of stress causing agents including hypoxia, metabolic stress and DNA damage [20]. ATF3 is also induced in times of physiological stress such as liver regeneration [21], brain seizure [22], ischemia-reperfusion of the heart and kidney [23], and nerve damage [24]. ATF3 has been shown to play a role in apoptosis and proliferation, two cellular processes critical for cancer progression [25-28]. Divergence in function of ATF3 between a pro-and anti-apoptotic factor in cancer models is dependent on both cellular model and state of malignancy [8,25,28]. Activation of ATF3 by a wide array of stress signalling pathways have been demonstrated including DNA repair pathway components p53 [29,30], the integrated stress response (ISR) that is principally activated by hypoxia and metabolic stress [31], and the MAPKinase cascades (SAPK/JNK, p38 and ERK) [32,33].

In this study we identify a novel target of the HDAC inhibitor M344, showing that treatment induced up-regulation of the stress inducible transcription factor ATF3. We show that M344 treatment can induce ATF3 expression at the protein and mRNA level in a panel of human derived cell lines. We also show that combination treatment with cisplatin and M344 could enhance induction of ATF3 compared with cisplatin alone. Likewise, M344 treatment increased the cytotoxic effects of cisplatin on the human cancer cell lines. Unlike cisplatin whose mechanistic induction of ATF3 was shown previously to be dependent on the MAPKinase pathways [19], ATF3 induction by M344 was found to be independent of the MAPKinase pathways and reliant on the ISR pathway. Finally, we correlated increased ATF3 expression with the enhanced cytotoxicity of M344 in combination with cisplatin utilizing ATF3 shRNA expressing cell lines. Taken together, this study identifies the pro-apoptotic factor, ATF3 as a novel target of HDAC inhibitors, as well as a novel factor regulating the co-operative effects of cisplatin and HDAC inhibitor induced cytotoxicity.

## Material and Methods

### Tissue Culture

The A549, PC3, and MCF-7 cell lines were obtained from American Type Culture Collection (ATCC; Rockville, MD). The SK-OV3 cell line was kindly provided by Dr. Barbara Vanderhyden, Ottawa Hospital Research Institute (OHRI), Ottawa, Canada. The MEFs used in this study were derived from heterozygote (+/-) and knockout mice (-/-) from an ATF4 murine model (kindly provided by D. Park, University of Ottawa, Ottawa, Ontario). All cell lines were maintained in DMEM supplemented with 10% fetal bovine serum (FBS; Medicorp, Montreal, Canada) and 100 units penicillin and 100 μg streptomycin (GIBCO, Burlington, ON)/ml of media. ATF4 (-/-) MEFs were maintained in DMEM containing 10% fetal bovine serum, 0.1 mM nonessential amino acids, 55 μM 2-mercaptoethanol, and 100 units penicillin and 100 μg streptomycin/ml of media. Cells were exposed to the HDAC inhibitor, 4-(Dimethylamino)-N-[7-(hydroxyamino)-7-oxoheptyl]-benzamide (M344) (Sigma, St. Louis, MI), or cisplatin (provided by the pharmacy at the Ottawa Hospital Regional Cancer Centre, Ottawa) alone or in combination with the p38 inhibitor SB203580 (Calbiochem, Gibbstown, NJ), JNK inhibitor, JNK inhibitor II (SP600125) (Calbiochem) or ERK inhibitor UO126 (Calbiochem) diluted in DMSO.

### 3-(4,5-Dimethylthiazol-2-yl)-2,5-Diphenyltetrazolium Bromide (MTT) Assay

In a 96-well flat-bottomed plate (Nunc, Naperville, IL) ~5,000 cells/150 μL of cell suspension were used to seed each well. The cells were incubated overnight to allow for cell attachment and recovery. Cells were treated with indicated drugs and incubated for 48 hrs at 37°C. Following treatment, 42 μL of a 5 mg/mL solution in PBS of the MTT tetrazolium substrate (Sigma) was added to each well and incubated for ~ 20 min at 37°C. The resulting violet formazan precipitate was solubilised by the addition of 82 μL of a 0.01 mol/L HCl/10% SDS (Sigma) solution, and allowed to further incubate at 37°C overnight. The plates were then analyzed on an MRX Microplate Reader from Dynex Technologies (West Sussex, UK) at 570 nm to determine the absorbance of the samples.

### Design and expression of small hairpin RNAs

The two 19 mer sequences targeting ATF3 mRNA are; #1-5'-GCCAAAGAATATTCCATTT-3' and #2- 5'-GGGAGGGCCTGCAGTGATT-3' to pSuper vector from Oligoengine small hairpin RNA (shRNA) (#1: nucleotides 1524-1542; GenBank accession number NM_001030287. #2: nucleotides 1270-1289; GenBank accession number NM_001030287) target sequence. As controls, we used the GFP-targeted oligonucleotide 5'CATGCGTCCACTCTTCCTC-3' with accession number NC_011521. These sequences were BLAST confirmed for specificity. The forward and reverse synthetic 60 nt oligonucleotides (Integrated DNA Technologies, Coralville, IA) were designed, annealed, and inserted into the *Bgl*II/*Hind*III sites of pSUPER.retro.puro vector, following the manufacturer's instructions (Oligoengine, Seattle, WA). These constructs express a 19 mer targeting two independent location within *ATF3 *mRNA or GFP (control shRNA) mRNAs. The retroviral packaging cell line, RetroPack PT67 (Clonetech Laboratories, Mountain View, CA) was used for stable virus production according to the manufacturer's instructions. Briefly, packaging cells were transfected with ATF3-shRNA plasmids#1, #2 or GFP-shRNA, using FuGENE^® ^HD Transfection Reagent (Roche, Mississauga, ON). After generation of stable clones and determination of viral titre, A549 cells were infected with viral supernatant using 4 μg/ml polybrene. Stable transfected clones expressing shRNAs were selected using 3 μg/ml puromycin.

### Western Blot Analysis

Cells plated at 0.7 ×10^6 ^per 60 mm dish were allowed to grow overnight and treated with indicated drug for 24 hrs. Protein samples were collected in RIPA buffer (50 mM Tris-CL pH 7.5, 150 mM sodium chloride, 1 mM EDTA, 1% Triton-X-100, 0.25% sodium deoxycholate, 0.1% SDS) containing 50 mM sodium fluoride, 1 mM sodium orthovanadate, 10 mM β-glycerolphosphate and 1× Protease Inhibitor Cocktail (Sigma-Aldrich, St. Louis, MO). Protein concentrations were assayed using Bio-Rad Protein Assay (Mississauga, Ontario, Canada) and a Biomate 3 Spectrophotometer (Thermo Fisher Scientific, Waltham, MA). Protein extracts representing 40 μg were separated on a 10% SDS-PAGE gel and electrophoretically transferred to a polyvinylidene difluoride membrane (Immobilon-P, Millipore, Billerica, MA). Membranes were blocked in 5% skim milk powder in Tris-buffered saline containing 10% Tween-20 (TBS-T) for 1 hr at room temperature followed by incubation with primary antibody diluted in 5% skim milk in TBS-T with shaking overnight at 4°C. Polyclonal antibody ATF3 was purchased from Santa Cruz, Santa Cruz, CA. Monoclonal anti-actin was purchased from Sigma-Aldrich, St. Louis, MO. Polyclonal antibody to PARP was purchased from Cell Signalling Technology, Beverly, MA. Polyclonal antibodies against HSP27 and phospho-HSP27 (Ser78) were purchased from Stessgen, Ann Arbor, MI. Following washes in TBS-T, blots were incubated with the appropriate HRP-labelled secondary antibody for 1 hr at room temperature. Visualization of protein bands was performed using the Supersignal West Pico Chemiluminescent Substrate (Pierce, Rockford, IL) exposed on Kodak film in a Konica Minolta SRX-101A tabletop processor.

### RT-RNA isolation and RT-PCR

MCF-7 cells plated at 0.8 ×10^6 ^cells per 10 cm dish were incubated at 37°C overnight. The next day cells were treated with either with M344, cisplatin or their combination for 24 hrs. Total RNA was extracted using the RNeasy1 kit (Qiagen, Mississauga, ON). RNA concentrations were quantified using a NanoDrop ND-1000 spectrophotometer (NanoDrop, Wilmington, DE). One microgram of total RNA was reverse-transcribed to complementary DNA for quantitative, real-time, reverse-transcriptase polymerase chain reaction (RT-PCR) as previously described [34]. The Applied Biosystems AB 7500 Real-Time PCR system (Applied Biosystems, Foster City, CA) was used to detect amplification. Real-time PCR reactions were carried out in a total volume of 25 μl that contained 2.5 μl of synthesized cDNA (42 ng), 1.25 μl of TaqMan Gene Expression Assay Primer/Probe (20× (Applied Biosystems, ATF3, HS00231069), 12.5 μl of TaqMan Universal PCR Master Mix (2× (Applied Biosystems, 4304437) and 8.75 μl of RNase-free water for ATF3 expression. The endogenous control for ATF3 was the housekeeping gene, human GAPDH (20× (Applied Biosystems, HS4333764-F). Amplification conditions were 95°C for 5 min, 40 PCR cycles at 95°C for 15 sec and 60°C for 1 min. Three independent experiments were performed to determine the average gene expression and standard deviation.

### Chromatin Immunoprecipitation (ChIP) Assay

Cells treated for 24 hrs in 10 cm dishes were fixed with 1% formaldehyde (BDH, VWR International, Mississauga, ON) for 20 min at room temperature in order to cross-link the DNA and protein. The cross-linking was quenched by adding glycine to a final concentration of 200 mM and incubating at room temperature for 5 min. Cells were then washed twice with ice-cold PBS and harvested in 1 mL cold PBS by centrifugation at 4°C for 5 min at 5,000 rpm. The pellet was resuspended in 90 μL lysis buffer (50 mM Tris-HCl pH 8.0, 10 mM EDTA pH 8.0, 1% SDS) supplemented with 1× Protease Inhibitor Cocktail (Sigma-Aldrich), 1 mM 1,4-dithio-DL-threitol (DTT) (Sigma-Aldrich), and 1 mM phenylmethylsulfonyl fluoride (PMSF) (Sigma-Aldrich). The lysates were sonicated using a Sonicator 3000 (Misonix, Farmingdale, NY) at power setting #1 for a total of 3 min on ice with 10 sec on/off pulses to shear the DNA to an average size of 300 to 1000 base pairs. Sonicated lysates were cleared of debris by centrifugation for 15 min at 14, 000rpm at 4°C. Input controls were removed from each sample and stored at -20°C. Sonicated lysates were divided into negative controls and samples, then diluted 10-fold with dilution buffer (20 mM Tris-HCl pH 8.0, 150 mM NaCl, 2 mM EDTA pH 8.0, 1% Triton X-100) supplemented with 1× Protease Inhibitor Cocktail (Sigma-Aldrich), 1 mM DTT (Sigma-Aldrich), and 1 mM PMSF (Sigma-Aldrich). Positive sample cell lysates were immunoprecipitated by overnight rotation at 4°C with rabbit anti-acetyl H4 (1:200, Millipore) primary antibody. Negative controls were incubated overnight with rotation at 4°C in the absence of primary antibody.

Immune complexes were collected by 2 hr rotation at 4°C with the addition of 40 μL of protein A agarose/salmon sperm DNA 50% slurry (Millipore) to both samples and negative controls. The agarose beads/immune complexes were then pelleted gently by centrifugation for 1 min at 3, 000 rpm at 4°C. The beads were washed with 1 mL of the following buffers by rotation for 10 min at 4°C, then pelleted gently by centrifugation for 1 min at 3,000 rpm at 4°C, discarding the supernatant following each wash: Buffer A (low salt; 0.1% SDS, 1% Triton X-100, 20 mM Tris-HCl pH 8.0, 2 mM EDTA pH 8.0, 150 mM NaCl) once, Buffer B (high salt; 0.1% SDS, 1% Triton X-100, 20 mM Tris-HCl pH 8.0, 2 mM EDTA pH 8.0, 500 mM NaCl) once, Buffer C (1% NP-40, 1% sodium deoxycholate, 20 mM Tris-HCl pH 8.0, 1 mM EDTA pH 8.0, 0.25 M LiCl) once, TE washing buffer (10 mM Tris-HCl pH 8.0, 1 mM EDTA pH 8.0) twice. Freshly prepared elution buffer (1% SDS, 100 mM NaCHO_3_) was added to all samples (input and negative controls, and samples) to a final volume of 400 μL and samples were rotated at room temperature for 30 min. The agarose beads were removed from the samples by centrifugation for 1 min at 3,000 rpm.

The DNA-protein cross-linking was reversed by overnight incubation with 5 μL proteinase K (20 mg/mL, Roche Diagnostics, Laval, QC, CAN) at 65°C. The DNA was purified using a QiaQuick PCR Purification Kit (Qiagen) according to the manufacturer's instructions. Purified DNA was eluted in 50 μL ddH_2_O and samples were stored at -80°C. Conventional PCR was performed with amplification conditions as follows; 95°C for 2 min, 40 PCR cycles of 95°C for 30 sec, 58°C for 30 sec, 72°C for 30 sec, and finally 72°C for 5 min. The binding of acetyl H4 to the ATF3 and p21 proximal promoter regions were determined using the following primer pairs: ATF3, forward-5' CCGAACTTGCATCACCAGTGC, reverse-5' GAGCTGTGCAGTGCGCGCC; p21, forward-GGTGTCTAGGTGCTCCAGGT, reverse -GCACTCTCCAGGAGGACACA [35]. PCR products were resolved on 1.6% agarose gels.

## Results

### HDAC inhibition induces ATF3 expression and enhances cisplatin cytotoxicity

**Figure 1:**
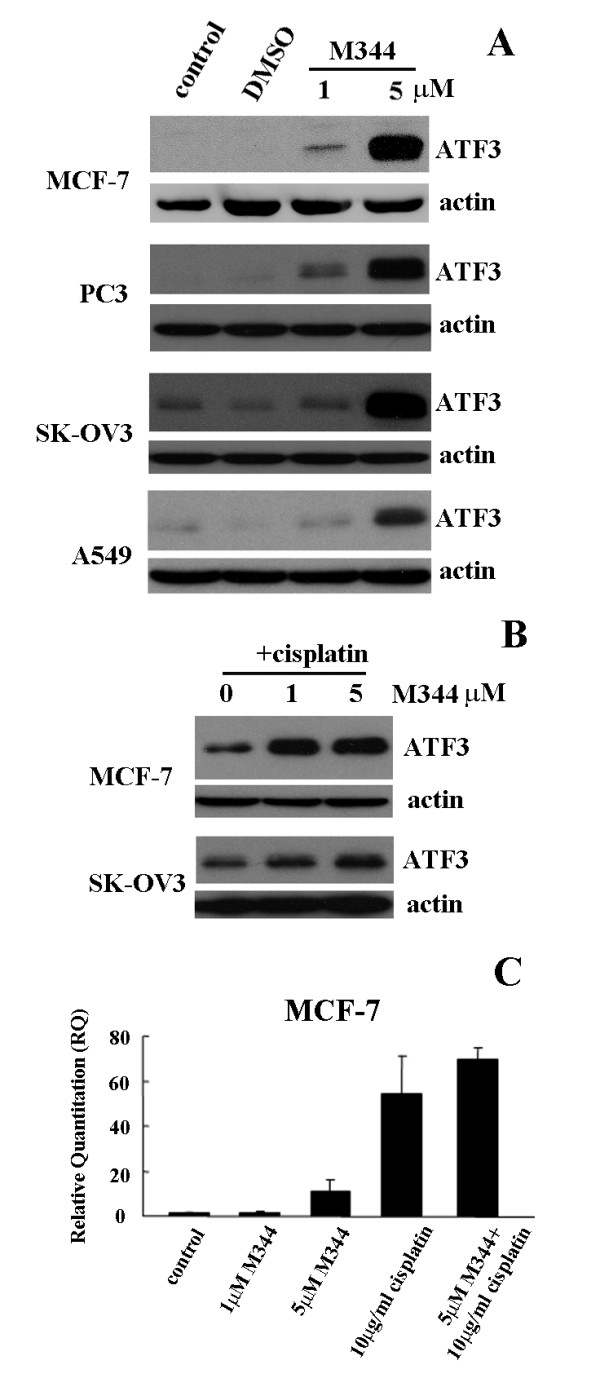
**M344 induces ATF3 and enhances its expression in combination with cisplatin treatment**. A, ATF3 protein expression levels following 24 hr treatment with control, DMSO vehicle, and, low and high doses of M344 (1 and 5 μM) in MCF-7, PC3, SK-OV3 and A549 cell lines. B, ATF3 protein expression levels following 24 hrs treatment with cisplatin (10 μg/ml), or cisplatin in combination with M344 (1 and 5 μM) in MCF-7 and SKOV-3 cell lines. In all blots actin is used as a loading control. C, ATF3 mRNA quantified by quantitative RT-PCR of MCF-7 cells comparing untreated treated control, DMSO vehicle, M344 (1 and 5 μM), cisplatin (10 μg/ml), or cisplatin in combination with M344 (5 μM) for 24 hrs. Error bars are representative of quantified mRNA from three independent experiments normalized to GAPDH expression.

**Figure 2:**
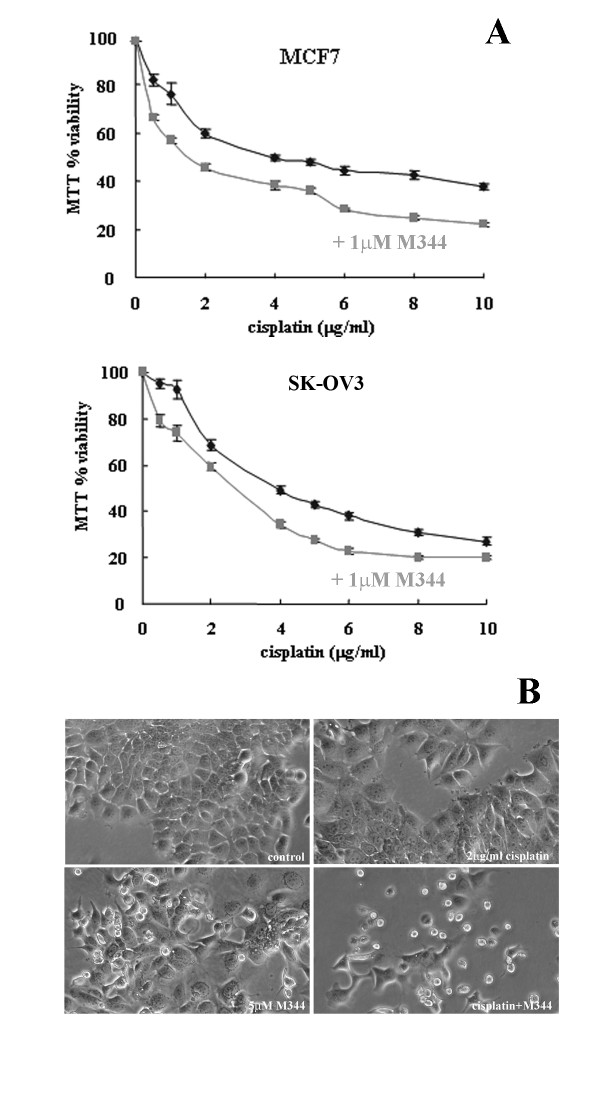
**M344 enhances the cytotoxicity of cisplatin**. A, MCF-7 and SK-OV3 cell lines treated with cisplatin (0-10 μg/ml) in the presence (grey) or absence (black) of M344 (1 μM) for 48 hrs was assessed for cell viability employing the MTT assay. Data is represented as a percentage of MTT activity where untreated cells were taken to be 100%. Error bars are representative of six replicates of two independent experiments. B, Phase contrast images of MCF-7 cells treated with no treatment (control), cisplatin (2 μg/ml), M344 (1 μM) and cisplatin and M344 in combination for 48 hrs.

We have recently demonstrated that ATF3 expression plays a role in cisplatin induced cytotoxicity [19]. Given the emerging role of HDAC inhibitors as anti-cancer agents, we evaluated whether ATF3 also regulates their activities. Indeed we found that M344 treatment, a potent pan HDAC inhibitor, could affect ATF3 expression following 24 hrs treatment. The higher (5 μM) dose of M344 in a panel of human derived cancer cell lines, MCF-7 (breast adenocarcinoma), PC3 (prostate carcinoma), SK-OV3 (ovarian carcinoma), and A549 (lung carcinoma) demonstrated consistent up-regulation of ATF3 protein expression (Figure 1A). Since our previous work had shown that cisplatin could also induce ATF3 expression, we evaluated ATF3 expression following combinational treatment with M344 and cisplatin. M344 treatment (1 and 5 μM) in combination with cisplatin (10 μg/ml) for 24 hrs enhanced induction of ATF3 compared with cisplatin treatment alone as determined by Western blot analysis (Figure 1B). M344 induction of ATF3 expression was also evaluated at the mRNA level in the MCF-7 cell line and found to be similarly induced under these experimental conditions (Figure 1C). Differences in ATF3 mRNA expression, although not statistically significant likely due to high variability of transcript induction between experiments, was generally additive in combination treatments compared with M344 and cisplatin treatment alone (Figure 1C). Since it has been shown that HDAC inhibitors can enhance the cytotoxicity of cisplatin, we confirmed this previous observation in the MCF-7 and SK-OV3 cell lines where combination treatment lead to approximately 20% increased cytotoxicity compared with cisplatin treatment alone (Figure 2A) as measured by the MTT cell viability assay. The observed enhanced cytotoxicity was also demonstrated by cell imaging following either cisplatin, M344 alone, or in combinational treatment in the MCF-7 cell line for 48 hrs (Figure 2B). A low dose of cisplatin was used (2 μg/ml) which does not induce significant cytotoxicity in the MCF-7 cell line (Figure 2A, top panel) however, following combination treatment with M344 (5 μM) enhanced cytotoxicity was clearly evident in the corresponding phase contrast images (Figure 2B). In summary, these data demonstrate that M344 is a novel inducer of ATF3 and an enhancer of ATF3 induction when in combination with cisplatin treatment. Increased ATF3 expression mediated by combinational treatment correlates with increased cytotoxicity compared with cisplatin alone.

### ATF3 induction by M344 is regulated by the Integrated Stress Response

**Figure 3:**
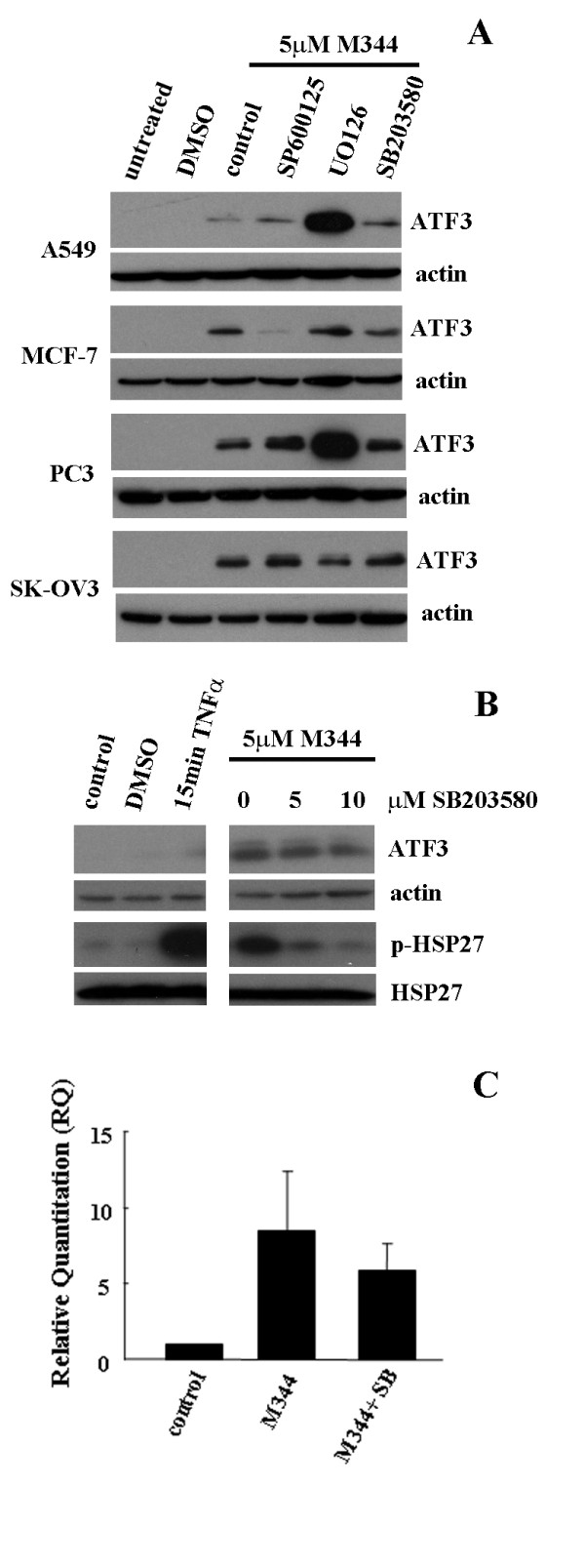
**ATF3 induction by M344 is independent of MAPKinase pathways**. A, A549, MCF-7, PC3 and SKOV-3 cells untreated (control), and treated with DMSO, or M344 (5 μM) for 24 hrs in the absence (control) or presence of MAPKinase pathway inhibitors SP600125 (50 μM), UO126 (25 μM), and SB203580 (10 μM) and analyzed by Western blotting for ATF3 and actin. B, MCF-7 cells untreated (control), treated with DMSO, TNFα for 15 min, or with M344 (5 μM) in the absence or presence of p38 inhibitor, SB203580 (5 and 10 μM) for 24 hrs were analyzed for ATF3, actin, phospho-HSP27 (p-hsp27), and total HSP27 expression by Western blotting. C. ATF3 mRNA quantified by quantitative RT-PCR of MCF-7 cells untreated (control), treated with M344 (5 μM) or M344 in the presence of SB203580 (10 μM) for 24 hrs. Error bars are representative of quantified mRNA from three independent experiments.

Next, we evaluated a number of cell signalling pathways that are known regulators of ATF3 expression to determine the mechanism of induction of ATF3 by M344. Our previous work had identified the MAPKinase pathways as mediators of ATF3 induction by cisplatin. Similarly, other groups had shown the involvement of MAPKinase pathways in mediating ATF3 induction through other stress inducing agents [32]. We evaluated the role of all the MAPKinase pathways using inhibitors to the JNK (SP600125), and ERK (UO126) as well as p38 (SB203580) pathways in all the cell lines used in this study. Unlike our previous data which showed that all inhibitors to these pathways could down regulate the induction of ATF3 by cisplatin consistently in all the same cell lines (MCF-7, PC3, SKOV-3 and A549), these inhibitors did not affect ATF3 induction by M344 treatment. This data essentially eliminates the MAPKinase pathways as regulators of ATF3 induction by M344 (Figure 3A). Although, decreased expression of ATF3 was observed following M344 treatment in the presence of JNK inhibitor in the MCF-7 cell line and ERK inhibitor in the SKOV-3 cell line, lack of consistency between cell lines allows us to conclude that MAPKinase pathways are likely not involved in mediating ATF3 induction by M344. In contrast, the ERK pathway inhibitor, UO126, could increase ATF3 expression when treated in combination with M344 on the A549 and PC3 cell lines (Figure 3A). Since ATF3 is a known stress inducible gene, the combination of M344 and inhibition of the ERK pathway, whose function is to mediate cell growth and differentiation, may specifically induce higher levels of ATF3 as a stress responsive cellular event. Of note in these cell lines, the inhibitors tested consistently inhibited ATF3 induction by cisplatin indicating a role for these MAPKinase cascades in cisplatin [19] but not M344 induction of ATF3 expression.

To rule out the involvement of the p38 MAPKinase pathway which we had previously shown had the most significant role in ATF3 induction by cisplatin, we more rigorously analyzed the role of the p38 MAPKinase pathway in M344 induction of ATF3. To determine the involvement of the pathway in mediating M344 induction of ATF3 the p38 specific inhibitor, SB203580 (SB), was utilized at increasing doses in the presence of M344 treatment for 24 hrs in the MCF-7 cell line. The pathway was effectively down regulated following inhibitor treatment in a dose dependent manner as measured by the phosphorylation status of heat shock protein 27 (HSP27), a downstream effector of the p38 pathway, however ATF3 expression was unaffected (Figure 3B). Controls included no treatment, DMSO was used as a control for the M344 vehicle, and TNFα as a positive control for p38 activation. To confirm this observation we also determined the mRNA expression of ATF3 following M344 treatment in the absence and presence of the p38 pathway inhibitor in the MCF-7 cell line and found no significant difference in ATF3 expression between treatments (Figure 3C). Taken together, these data confirm a MAPKinase independent mechanism as a mediator of ATF3 induction by M344.

**Figure 4:**
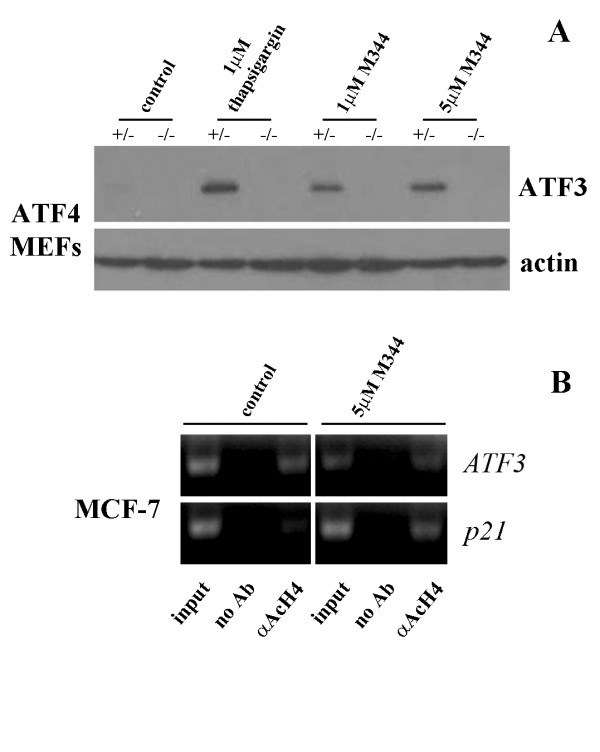
**M344 induced ATF3 is mediated through the Integrated Stress Response**. A, Western blot analysis for ATF3 and actin in ATF4 (+/-) and (-/-) MEFs treated with solvent control, thapsigargin (1 μM), M344 (1 and 5 μM) for 24 hrs. B, Chromatin Immunoprecipication (ChIP) assay of chromatin/DNA complexes isolated from MCF-7 cells treated with solvent control or M344 (5 μM) for 24 hrs using acetylated Histone 4 (AcH4) antibody and analyzing by PCR for presence of ATF3 or p21 promoter regions.

Previously our laboratory had identified lovastatin, a potent inhibitor of mevalonate synthesis, as an inducer of the ISR pathway and subsequent mediator of lovastatin-induced apoptosis [34]. Downstream effectors of the ISR pathway include members of the ATF family of transcription factors, ATF4 and its downstream target ATF3 [31]. Therefore, we looked at the potential involvement of the ISR pathway, and specifically ATF4, as a mediator of ATF3 induction by M344. We tested the ability of M344 to induce ATF3 expression in immortalized ATF4 heterozygous (+/-) or null (-/-) MEFs, the upstream inducer of ATF3 expression in the ISR pathway. Using thapsigargin, a well established inducer of the ISR, as a positive control [36], we show in Figure 4A that the absence of ATF4 completely inhibits ATF3 induction by M344 revealing an ISR dependent mechanism.

Since it has been shown that HDAC inhibition can mediate induction of genes by directly influencing the acetylation of histones surrounding the gene thus promoting transcription, we performed a ChIP assay to evaluate the association between acetylated Histone 4 (AcH4) and the ATF3 promoter. Chromatin was isolated from the MCF-7, and PC3 cell lines following treatment with solvent control or M344 at 1 and 5 μM doses. Chromatin- protein complexes were pulled down with an antibody against AcH4 and the DNA was assessed for the presence of the ATF3 promoter region. In both cell lines, pull down with AcH4 antibody in the untreated cells yielded the presence of the ATF3 promoter without significant enhancement with M344 treatment (Figure 4B). Following M344 treatment, ATF3 gene expression was increased as compared with control cells (see Figure 1), however, ATF3 promoter expression associated with AcH4 was not increased as compared with control (Figure 3B) suggesting the induction of ATF3 by M344 is independent of histone acetylation association with the ATF3 gene promoter. As a control, M344 treatment induced AcH4 at the p21 promoter, a well established target of HDAC inhibition whose expression is up-regulated through promoter histone acetylation [35]. These data suggest the induction of ATF3 by M344 to be indirect and related to its activation and induction of effectors of the ISR.

### ATF3 regulates, in part, the enhanced cytotoxicity of cisplatin and M344

**Figure 5:**
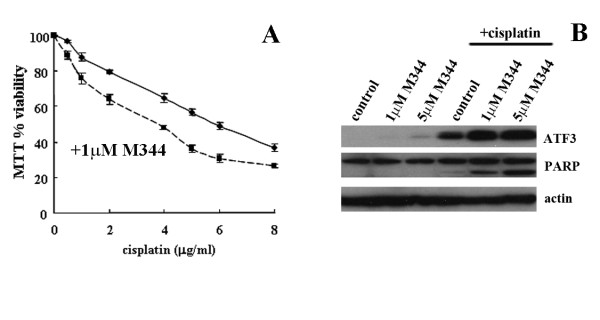
**ATF3 mediates, in part, the enhanced cytotoxicity between M344 and cisplatin**. A, A549 cells treated with cisplatin alone (0-10 μg/ml), or with M344 (1 μM) for 48 hrs were assessed for cell viability as measured by MTT activity. Data is represented as a percentage of MTT activity where untreated cells were taken to be 100%. Error bars are representative of six replicates of two independent experiments. B, A549 cells treated with solvent control, M344 alone (1 and 5 μM), cisplatin (2 μg/ml) alone or in combination with M344 (1 and 5 μM) were analyzed by Western blotting for ATF3, PARP and actin.

**Figure 6:**
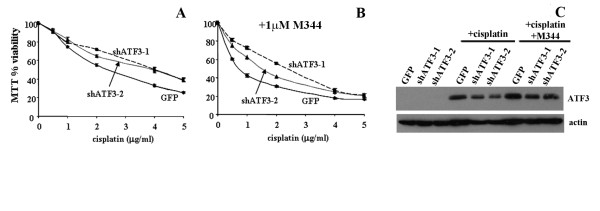
**Targeting ATF3 expression inhibits M344 and cisplatin co-operative cytotoxicity**. A, A549 cells stably expressing GFP or shATF3-1 and shATF3-2 were treated with cisplatin (1-5 μg/ml) for 48 hrs and analysed for MTT activity as above. B, A549 cells stably expressing GFP or shATF3-1 and shATF3-2 were treated with cisplatin in the presence of M344 (1 μM) for 48 hrs and analysed for MTT activity as above. * Statistical analysis, Paired T-test P < 0.005 between GFP and both shATF3 sub-lines. C, A549 cells stably expressing GFP (negative control), or short hairpin RNA against two separate ATF3 mRNA regions (shATF3-1) and (shATF3-2) were untreated, treated with cisplatin (10 μg/ml) or cisplatin in combination with M344 (5 μM) for 24 hrs and analyzed by Western blotting for ATF3 and actin expression.

To determine whether ATF3 expression affects the enhanced cytotoxicity observed between cisplatin and HDAC inhibitor treatments, we evaluated ATF3 induction by M344 and cisplatin combination treatment in the A549 cell line. As demonstrated for the MCF-7 and SK-OV3 cells in Figure 2A, the combined drug treatments in A549 cells was associated with increased cytotoxicity compared to cisplatin treatment alone as analyzed by the MTT cell viability assay (Figure 5A). Furthermore, the combined treatment of cisplatin and M344 also resulted in enhanced ATF3 expression as compared with cisplatin and M344 alone as observed by Western blotting (Figure 5B). Likewise, PARP cleavage, a marker of apoptosis, was observed to increase following cisplatin and M344 treatment in combination compared with M344 and cisplatin treatment alone (Figure 5B). To further elucidate the role of ATF3 in enhanced cytotoxicity by HDAC inhibitors in combination with cisplatin, we expressed shRNA targeting ATF3 in the A549 cell line. To determine the role of ATF3 expression in drug-mediated cytotoxicity, GFP (negative control), shATF3 1 and 2 stably expressing cell lines that target two distinct sequences of the ATF3 gene were treated with cisplatin alone (Figure 6A) or cisplatin in combination with M344 (Figure 6B) and analyzed by the MTT assay. As previously reported [19], the shRNA expressing ATF3 targeted A549 cell lines showed attenuated cisplatin induced cytotoxicity as compared with GFP control (Figure 6A). M344 (1 μM) treatment in combination with cisplatin enhanced cell cytotoxicity as compared with cisplatin alone in all cell lines (Figure 6A and 6B). Cytotoxicity was also attenuated in both of the shATF3 cell lines compared with GFP control when treated with cisplatin in combination with M344 (Figure 6B). Cisplatin (2 μg/ml) and M344 (1 μM) combined treatment enhanced ATF3 expression in the GFP control while ATF3 induced expression was reduced in the shRNA targeting ATF3 A549 cells with these treatments (Figure 6C). Since the inhibition of ATF3 expression inhibits the enhanced cytotoxicity of this drug combination, these data provide evidence that ATF3 plays a role in mediating the enhanced cytotoxic response.

## Discussion

In this study, we identified ATF3 as a novel consistently inducible target of HDAC inhibitor treatment in a panel of human derived cancer cell lines both at the protein and mRNA level. Similarly in a very recent study, ATF3 was identified as one of a number of genes induced following a genetic screen of an HDAC inhibitor in sensitive colon cancer cell lines although the mechanism of induction was not characterized [37]. This is the first study to characterize this regulation in multiple cancer cell lines as well as address the mechanism of HDAC inhibition induced ATF3 expression. Regulators of ATF3 expression include the MAPKinase pathways as well as ISR activation. In M344 treatments, MAPKinase pathways, including the p38, ERK and JNK pathways, did not play a role in the induction of ATF3 expression by this HDAC inhibitor. In contrast, we have recently demonstrated that these same MAPKinase pathways regulate cisplatin induced ATF3 expression. To address the role of MAPKinases, we employed specific inhibitors to these pathways in a cancer cell line panel and found no consistent inhibition of M344 mediated ATF3 induction. Interestingly, we observed an up regulation of ATF3 expression when treating A549 and PC3 cell lines with M344 in combination with the ERK inhibitor UO126. Combination treatment of the MEK/ERK inhibitor UO126 and the HDAC inhibitor SAHA lead to increased apoptosis in leukemia cell lines [38], however, ATF3 levels were not assessed [38].

In this study, we provide evidence for the involvement of the ISR pathway as mediator of M344 induction of ATF3. M344 induced expression of ATF3 was completely abolished in ATF4 (-/-) MEFs implicating an ISR dependent mechanism downstream of ATF4. In accordance with this finding, the endoplasmic reticulum chaperone protein glucose-regulated protein 78 (GRP78) was recently identified as a non-histone target of SAHA, whose action leads to dissociation of GRP78 and its client protein, double-stranded RNA-activated protein-like ER kinase (PERK), and subsequent activation of the ISR through the induction of the endoplasmic reticulum stress response including activation of ATF4 [39]. Since ATF3 is a known effector of the ISR pathway downstream of ATF4 [36], our finding that M344 induces ATF3 may be mediated by HDAC inhibitor mediated acetylation of GRP78. Furthermore, we also demonstrated through ChIP assay of the ATF3 promoter that levels of acetylated histone H4/chromatin were independent of M344 suggesting the induction of ATF3 was not the result of increased histone acetylation at the ATF3 promoter.

A role for ATF3 in tumourigenesis has been implicated through its documented role as an apoptotic factor in cancer models, whose mechanism may be related to ATF3's role in transcriptional regulation of a number of regulators of apoptosis and cell proliferation including pro-apoptotic factor, GADD153/CHOP and cell cycle factor, cyclin D1, respectively [40,41]. Depending on the cell type and the type and severity of the cell stressor, ATF3 has been implicated as both a proto-oncogene and tumour suppressor. Examples of ATF3 as a pro-apoptotic include an ATF3 over-expression model which lead to inhibition of proliferation and induced cell cycle arrest in human cancer cells [27], and loss of ATF3 in a Ras transformed model which resulted in higher proliferation rates and increased G1 to S phase transition efficiency [28].

As stated, HDACs catalyze the removal of acetyl groups from histones resulting in chromatin condensation and transcriptional repression [1,2]. HDAC inhibitors reverse this transcriptional silencing of genes, including tumour suppressors [1,2]. Coupled with their ability to induce such anti-cancer cellular processes as cell cycle arrest, apoptosis, and disruption of angiogenesis, HDAC inhibitors have been studied for their potential as cancer therapeutic agents [1-4]. Cisplatin, on the other hand, is considered a DNA-damaging anticancer drug forming different types of bi-functional adducts in reaction with cellular DNA. The final cellular outcome of DNA adduct formation is generally apoptotic cell death, thought to occur through halting of cellular processes such as replication and transcription leading to prolonged G2 phase cell-cycle arrest and deregulation of signal transduction pathways involved in growth, differentiation, and stress responses [7]. There is a growing body of evidence that demonstrates that HDAC inhibitors can enhance the anticancer activity of a variety of chemotherapeutic drugs, including cisplatin [12-17,42].

Previous reports have attempted to identify the factors related to HDAC inhibitors ability to enhance cisplatin induced cell death including decreasing the levels of the antioxidant intracellular reduced glutathione or the involvement of the endoplasmic reticulum stress response as a mediator of the enhancement of cytotoxicity in the same cancer model [43]. Up-regulation of the expression by HDAC inhibitors in apoptosis associated proteins p53, BID, cytochrome c and caspase-3 have also been proposed as targets of HDAC inhibitors that can enhance cisplatin-induced cytotoxicity [16]. In this study we identified the transcription factor ATF3 as a mediator of enhanced cisplatin induced cytoxicity by HDAC inhibition. Identification of the specific pathway(s) of apoptotic cell death related to ATF3's role as mediator of enhanced cytotoxicity by combinational treatment merits further investigation.

## Abbreviations

ATF: activating transcription factor; HDAC: Histone Deacetylase; MAPKinase: mitogen-activated protein kinase; ISR: integrated stress response; MEFs: murine embryonic fibroblasts; cisplatin: cis-Diamminedichloroplatinum(II).

## Competing interests

The authors declare that they have no competing interests.

## Authors' contributions

CSG carried out the molecular and cell viability studies, participated in the design of the study and drafted the manuscript. AO carried out the ChIP studies and helped to draft the manuscript. JD conceived of the study, participated in its design and coordination and helped to draft the manuscript. All authors read and approved the final manuscript.
